# Long non-coding RNA LOC285194 inhibits proliferation and migration but promoted apoptosis in vascular smooth muscle cells via targeting miR-211/PUMA and TGF-β1/S100A4 signal

**DOI:** 10.1080/21655979.2020.1788354

**Published:** 2020-07-03

**Authors:** Shaochun Wang, Ping Li, Gang Jiang, Jinping Guan, Dong Chen, Xiaoying Zhang

**Affiliations:** a Department of Emergency Medicine, The Affiliated Hospital of Qingdao University, Qingdao, Shandong, China; b Ultrasound, The Affiliated Hospital of Qingdao University, Qingdao, Shandong, China; c Radiology, The Affiliated Hospital of Qingdao University, Qingdao, Shandong, China; d Emergency Surgery, Ultrasound, The Affiliated Hospital of Qingdao University, Qingdao, Shandong, China; e General Surgery, The Affiliated Hospital of Qingdao University, Qingdao, Shandong, China

**Keywords:** Vascular smooth muscle cell, apoptosis, proliferation, migration, long non-coding RNA LOC285194, miR-211, PUMA, S100A4

## Abstract

Long non-coding RNA LOC285194 (LOC285194) has reported to regulate vascular smooth muscle cells (VSMCs) proliferation and apoptosis *in vitro* and *in vivo*. Here we aimed to determine the role of LOC285194 in the proliferation, migration and apoptosis of VSMCs and its underlying mechanisms. A7r5 cells were transfected with Lv-LOC285194 or control Lv-NC for 24–72 h, or small interfering RNA targeting S100A4 (S100A4 siRNA) for 24–48 h, or co-transfected with Lv-LOC285194 and PUMA siRNA for 72 h, or treated with miR-211 inhibitor or co-transfected with Lv-LOC285194 and miR-211 mimics for 72 h. A7r5 cells were also treated with transforming growth factor – β(TGF-β) (5 ng/ml) after Lv-LOC285194 transfection for 24 h. The relationship between LOC285194 and TGF-β was confirmed using luciferase reporter assay. Cell proliferation and cell apoptosis were analyzed by Cell Counting Kit-8 (CCK-8) assay, ELISA and TUNEL staining. LOC285194 and miR-211 expression were detected by qPCR assay. S100A4, pro-apoptotic and anti-apoptotic protein were detected by Western blot assay. LOC285194 inhibited cell proliferation, invasion and migration and promoted cell apoptosis accompanied by upregulation of PUMA and downregulation of miR-211 and S100A4. Targeting PUMA reversed the effect of LOC285194 on cell apoptosis and proliferation. miR-211 mimic inhibited LOC285194-induced PUMA upregulation and decreased LOC285194-induced cell apoptosis. TGF-β (5 ng/ml) treatment reversed S100A4 siRNA or LOC285194-induced S100A4 expression. Luciferase reporter assay showed that TGF-β was the target of LOC285194. LOC285194 inhibits proliferation and promoted apoptosis in vascular smooth muscle cells via targeting miR-211/PUMA signal; In addition, LOC285194 decreased cell invasion and migration by targeting TGF-β1/S100A4 signal.

## Introduction

Atherosclerotic cardiovascular disease (ASCVD) is an inflammatory disease characterized by extensive arterial wall matrix protein degradation, resulting in endothelial injury occurring throughout the course of the disease [1]. ASCVD and its clinical manifestations, such as myocardial infarction (MI) and ischemic stroke, are the leading cause of morbidity and mortality throughout the world [2]. Although preventive drug therapies reduce the relative risk of cardiovascular events in primary and secondary prevention patients, the absolute risk of subsequent ASCVD events remains high [2].

Vascular smooth muscle cell (VSMC) apoptosis plays an important role in vascular remodeling and in the pathophysiology of aneurysms, post-angioplasty restenosis and atherosclerosis [3]. During early atherogenesis and upon vascular injury, and aged VSMCs from rodents, cell proliferation is increased [4]. Reduced apoptosis or overproliferation of VSMCs accelerates the deposition of atherosclerotic plaques in the lining of blood vessels, and thicken intimal and induces vascular remodeling [5,6]. In addition, the presence of a large number of intimal VSMCs has been taken as evidence that VSMC migration from the media plays an important role in atherogenesis [7,8]. Therefore, inhibiting VSMC cell migration, proliferation and apoptosis may represent promising therapies for protecting blood vessels against atherosclerosis.

Long non-coding RNAs (lncRNAs) recently have been implicated in many biological processes and diseases. We have recently reported that overexpression of long non-coding RNA LOC285194 (LOC285194) leads to decreased proliferation and increased apoptosis in HA-VSMC cells and vice versa. In addition, targeting LOC285194 promotes cell invasion and migration *in vitro* [9]. However, the mechanisms by which LOC285194 influences VSMC migration, proliferation and apoptosis remain unclear.

Liu et al. reported that exon 4, which harbors two miR-211 binding sites, inhibits tumor cell growth; loc285194-mediated growth inhibition is also in part due to specific suppression of miR-211 [10]. In addition, P53-up-regulated modulator of apoptosis (PUMA) was identified as a target gene of miR-211, and targeting miR-211 facilitated OGD/R-induced PC12 cell apoptosis *in vitro* by upregulation of PUMA and vice versa [11]. We suggested that LOC285194 induces VSMC cell apoptosis by LOC285194-miR-211-PUMA signals.

The transforming growth factor -β (TGF-β) signaling pathway is critical for promoting and maintaining the contractile phenotype of VSMCs [12], whereas the TGF-β-mediated contractile phenotype of VSMCs that acts through inhibiting cell migration [13,14]. S100A4 is a calcium-binding protein that has been shown to promote cancer progression and metastasis [15], enhance the motility of macrophages, neutrophils and leukocytes, and promote these inflammatory cells’ recruitment and chemotaxis to regulate inflammation and immune functions [16]. In many cancer cells, S100A4 is required for TGF-β1 effects on cell migration and invasion [17,18]. TGF-β induced myofibroblastic features in human dermal microvascular endothelial cells via induction of S100A4 expression [19]. TGF-β also mediating intestinal fibroblast migration via S100A4 upregulation [20]. We suggested that TGF-β- S100A4 signal might regulate VSMC migration. It has reported that the expression of LOC285194 inhibited the migration of CSCC cells in vitro through the inactivation of TGF-ß1 [21]. We suggested that LOC285194 induces VSMC cell migration by LOC285194- TGF-β- S100A4 signal.

In the present study, we investigated the role and underlying mechanisms of LOC285194 on VSMC migration, proliferation and apoptosis.

## Materials and methods

### Cell line and cell culture

The rat aortic VSMC line A7r5 cell was purchased from the European Collection of Cell Cultures through Shanghai Cell Research Institute (Shanghai, China). The cells were cultured in Dulbecco’s modified Eagle’s medium (DMEM) supplemented with 10% FBS, 100 U/mL penicillin and 100 U/mL streptomycin at 37°C temperature with 5% CO_2_.

### Immunoﬂuorescence staining

A7r5 cells were ﬁxed with 100% methanol and blocked with 0.1% Triton-X100 and 4% bovine serum albumin (BSA) with 10% goat serum in phosphate-buffered saline (PBS) for 60 mins at room temperature. Then the cells were incubated with primary anti-a-SM in PBS containing 1% BSA for 12 h at 4°C (1:200; Santa Cruz, Shanghai, China). The ﬂuorescence intensities were quantiﬁed using ImageJ software.

### Lentivirus production and infection

The pGCSIL-GFP vector containing a full-length sequence of LOC285194 or the control sequence were co-transfected into 293 cells. The transfection was generated using Lipofectamine 2000 (Invitrogen) following the manufacturer’s instructions. After 24–72 h of infection, the infection efficiency was verified using Western blot.

### RNA interference

The murine S100A4 siRNA (S100A4 siRNA) sequence was 5′-UGA ACA AGA CAG AGC UCA Att-3′ (sense) and 5′-UUG AGC UCU GUC UUG UUC Att-3′ (antisense), and the non-targeting siRNA (NC siRNA) sequence was sense: 5′-UUCUCCGAACGUGUCACGUTT-3′ and antisense: 5′-ACGUGACACGUUCGGAGAATT-3′ were purchased from Life Technologies (Shanghai, China). A7r5 cells were transfected with a final concentration of 20 nM of siRNA for 6 h performing in Lipofectamine™ 2000 according to the manufacturer’s recommendations. After incubation for 6 h, the medium was replaced with the standard culture medium. After an additional 42 h incubation, cells were used for further experiments. The transfected colonies referred to as A7r5/S100A4 siRNA or A7r5/NC siRNA cells, respectively.

### Transfection with miR-211 mimics and inhibitors

The miR-211 mimics, miR-211 inhibitor and their scrambled miRNAs were purchased from GeneChem (Shanghai, China). The 100 nM miR-211 mimics and inhibitors were transfected into A7r5 cells using Lipofectamine RNAiMAX reagent (Invitrogen) as the manufacture’s instruction.

### TGF-β treatment

48 h after Lv-LOC285194 transfection, A7r5 cells were treated with TGF-β (5 ng/ml) for 24 h. The A7r5 cells without transfection were also treated with TGF-β (5 ng/ml) for 24 h. Further detection was carried out below.

### Dual-luciferase reporter assay

The dual-luciferase reporter vector containing the wild-type (WT) LOC285194 3′-UTR sequence was from OriGene, and QuickChange Site-directed Mutagenesis Kit (Stratagene) was used TGF-β-binding site mutation. The two constructs were termed WT (Gene-wild type) and MT (Gene-mutant). The fragments were cloned into the psiCHECK™-2 vector using Lipofectamine 2000 (Invitrogen) according to the manufacturer’s instructions. Luciferase assays were conducted by transfecting subconfluent A7r5 cells according to the manufacturer’s instructions. Cells were lysed 16 h after transfection, and lysates were analyzed using a dual-luciferase reporter assay kit (Promega) and normalized to that of firefly luciferase.

### Western blotting

Cell extracts were prepared in RIPA (radioimmunoprecipitation assay) buffer supplemented with Complete protease inhibitor cocktail (Roche). Equal amounts of cell extracts were resolved on acrylamide: bis-acrylamide gels and electroblotted onto PVDF membrane (Immobilon-P, Millipore) and probed with appropriate primary and HRP-conjugated secondary antibodies (Jackson ImmunoResearch). When necessary, membranes were stripped using Restore Western Blot Stripping Buffer (Thermo) and reprobed with anti-PUMA, anti-GAPDH, anti-S100A4, anti-MMP-2/9, anti-bcl-2, anti-bax and cleaved-caspase-3 (Santa Cruz, Shanghai, China). Unless otherwise specified, western blots were representative of n = 3.

### Quantitative PCR (qPCR)

Total RNA was extracted from cells using Tri-RNA Reagent (Takara, Dalian, China). Total RNA was reverse-transcribed to cDNA using the PrimeScript^TM^RT reagent Kit and random primers (Takara, Dalian, China). Pairs of primers were designed and optimized to quantify expression levels of miR-211 using quantitative PCR (qPCR). Results were analyzed using 2^−ΔΔCT^ method.

### Detection of cell viability by CCK-8 assay

After transfection, cells were digested with 0.25% trypsin for 1 min and counted by a hemocytometer. Cells were seeded in 96-well plates at a density of 5 × 10^3^ cells/well with three duplications and incubated at 37°C overnight. Then, the cells were treated according to the requirement of corresponding groups and further cultured for 24–96 h. After that, 10 μL of CCK-8 reagent was added to each well and incubated at 37°C for 2 h. The absorbance at 450 nm was determined and the cell viability was calculated.

### TUNEL assay

Terminal deoxynucleotidyl transferase dUTP nick end labeling (TUNEL) was done using a kit according to the manufacturer’s protocol (In Situ Cell Death Detection Kit, Shanghai, China). Briefly, the A7r5 cells (100 µl of 2 × 10^4^ cells) were added into each well of a 96-well plate and incubated overnight, followed by cell transfection (Lv-LOC285194 or control Lv-NC, or co-transfection with S100A4 siRNA). Then, the cells were cultured in conventional conditions for 72 h. The medium was discarded and the cells were fixed, the DNA nicks in apoptotic cells were labeled with fluorescein-conjugated nucleotides. Nuclei were stained with Hoechst. Apoptosis was semi-quantitatively evaluated by the ratio of TUNEL positive cell number divided by Hoechst positive cell number.

### Enzyme-linked immunosorbent assay for apoptosis

A7r5 cells were seeded on a 96-well plate at a density of 2 × 10^4^ cells/well and cultured in conventional conditions overnight. Then, the cells were transfected with Lv-LOC285194 or Lv-NC for 72 h, or co-transfection with S100A4 siRNA for 72 h. Cell apoptosis was detected using an enzyme-linked immunosorbent assay (ELISA) kit, according to the manufacturer’s protocol (Cell Death Detection ELISA, Roche Molecular Biochemicals).

### Wound healing assay

The Lv-LOC285194 or Lv-NC or S100A4 siRNA and control cells were cultured into six‐well plates (1–1.2 × 10^5^ cells/well) overnight. Then, the surface of cell monolayers was scratched with a 200-µL micropipette tip. The wounded cells were washed several times with PBS to eliminate debris. The cells were then grown in a serum-free medium for 24 or 48 hr before final images were acquired. Migration rates were calculated by measuring the distance traveled toward the center of the wound using an Olympus CKX41 inverted microscope coupled with a digital imaging system.

### Transwell assay

Transwell migration assay was performed by using 8.0-μm cell culture inserts (Millipore, Darmstadt, Germany) to test the migratory ability of A7r5 cells with or without Lv-LOC285194 or Lv-NC or S100A4 siRNA transfection. After incubation for 24 h at 37°C, cells on the upper surface of the membrane were scraped off. The invading cells on the membrane were fixed in 3.7% paraformaldehyde and stained using DAPI (Roche Diagnostics, Indiana, IN). Images were taken in 10 different fields for the sum of invading cells. Nuclei of migrated cells were counted in five high-power fields with a 20× objective.

### Statistical analysis

All quantitative data were obtained from at least three independent experiments. All analyses were performed with SPSS 22.0 (SPSS). Data were presented as the mean ± SEM. Student’s *t*-test and one-way ANOVA were used for statistical analysis. *p* < 0.05 were considered significant.

## Results

### Characterization of A7r5 cells

**Figure 1.**
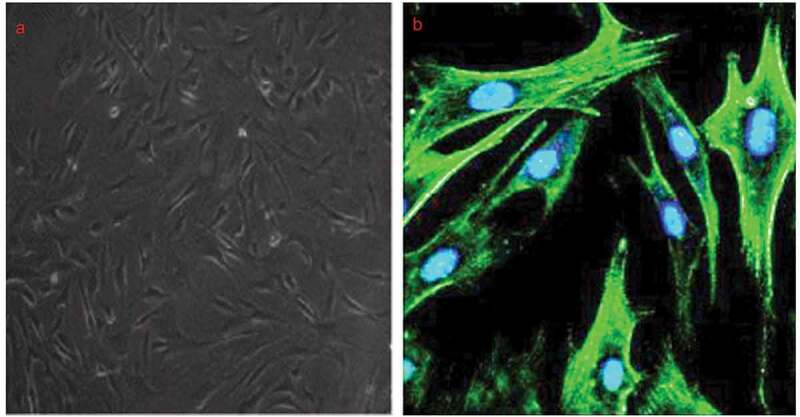
Identification of A7r5 cells obtained from European collection of cell cultures.

The A7r5 cells purchased from the European Collection of Cell Cultures were identified by α-SMA staining, a VSMC-specific marker and found that more than 98% of the cells were α-SMA-positive (Figure 1).

(a) Primary cultures of *A7r5* (phase contrast). (b) Co-staining of nuclei and α-smooth muscle actin (fluorescence/immunofluorescence)(200× magnification).

### LOC285194 inhibits proliferation and induces apoptosis *in vitro*

**Figure 2.**
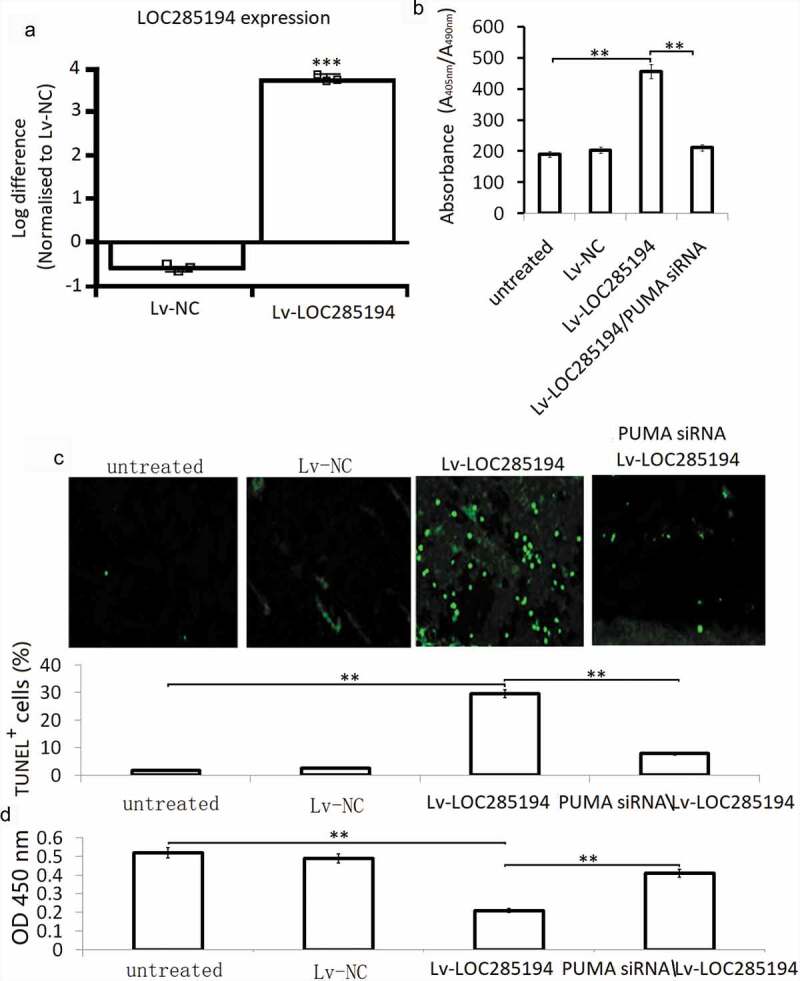
Effect of LOC285194 overexpression on proliferation and apoptosis in A7r5 cells *in vitro.*

A7r5 cells were infected with an Lv-LOC285194 or Lv-NC for 72 h for apoptosis assay using an ELISA-based method. The results showed that LOC285194 overexpression (Figure 2(a)) induced significant cell apoptosis in the A7r5 cells (Figure 2(b)). TUNEL staining also showed increased TUNEL positive cells in the Lv-LOC285194 overexpressing A7r5 cells (Figure 2(c)). We next investigated the influence of LOC285194 overexpression on the viability of A7r5 cells, compared with the Lv-NC, the Lv-LOC285194 group showed significant inhibition of cell viability (Figure 2(d)).

(a) A7r5 cells were infected with Lv-LOC285194 or Lv-NC for 72 h. (a) LOC285194 expression was detacted by qPCR assay; (b) A7r5 cells were co-infected with Lv-LOC285194/Lv-NC and PUMA siRNA, cell apoptosis was detected by ELISA. (c) cell apoptosis was detected by TUNEL staining. (d) cell proliferation was detected by CCK-8 assay; *p < 0.05; **p < 0.01; ***p < 0.001.

### LOC285194 induces apoptosis through upregulation PUMA *in vitro*

**Figure 3.**
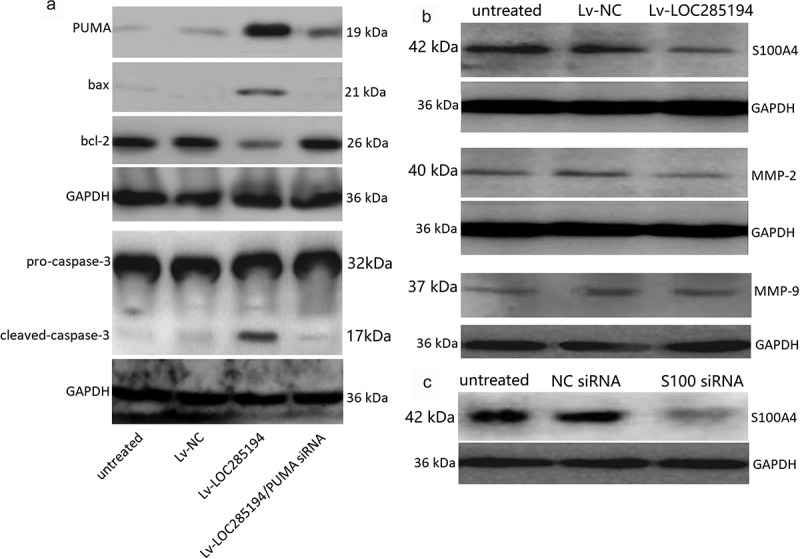
Effect of LOC285194 overexpression on apoptotic and metastatic protein expression *in vitro.*

A7r5 cells were co-transfection with PUMA siRNA and Lv-LOC285194 for 72 h. Lv-NC and NC siRNA were as the control. Lv-LOC285194 treatment alone upregulated PUMA, bax and activated caspase-3, and downregulated bcl-2 (Figure 3(a)). After co-incubated with PUMA siRNA and Lv-LOC285194 for 72 h, Lv-LOC285194-induced upregulation of PUMA, bax and activated caspase-3 was decreased and bcl-2 expression was upregulated (Figure 3(a)). Furthermore, targeting PUMA markedly decreased Lv-LOC285194-induced cell apoptosis (Figure 2(b,c)). Analysis of cell viability by CCK-8 assay, PUMA siRNA partly restored Lv-LOC285194-induced cell viability (Figure 2(d)), suggesting that LOC285194-induced apoptosis of A7r5 s cells was mediated by the activation of PUMA.

(a) A7r5 cells were infected with Lv-LOC285194 or Lv-NC for 72 h, pro- and anti-apoptotic protein expression were detected by Western blot assay. (b) A7r5 cells were infected with Lv-LOC285194 or Lv-NC for 72 h, S100A4, MMP-2/9 were detected by western blot assay; A7r5 cells were infected with S100A4 siRNA or NC siRNA for 24 h, S100A4 protein expression was detected by western blot assay.

### LOC285194 inhibits invasion and migration *in vitro*

**Figure 4.**
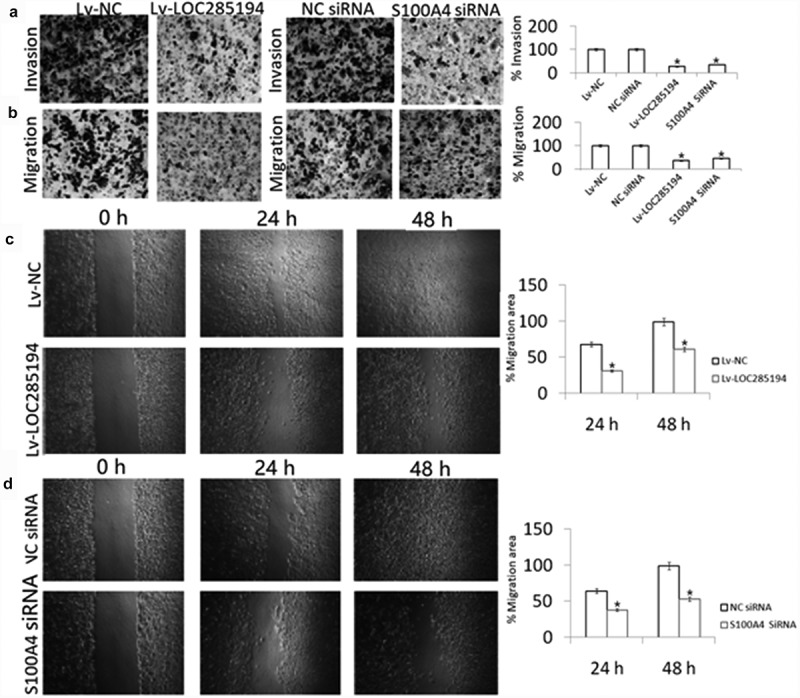
Effect of LOC285194 overexpression and S100A4 downexpression on invasion and migration *in vitro.*

A7r5 cells were transfection with Lv-LOC285194, Lv-NC, or negative control for 24 h. As shown in Figure 4(a–c), Lv-LOC285194 treatment decreased cell migration and invasion by wound healing assay and Transwell assays, suggesting that LOC285194 overexpression inhibits the A7r5 cells invasion and migration.

(a,b) A7r5 cells were infected with Lv-LOC285194 or S100A4 siRNA and its control for 24 h; cell invasion and migration were detected using Transwell assay; (c,d) A7r5 cells were infected with Lv-LOC285194 or S100A4 siRNA and its control for 24 h and 48 h; cell migration was detected using Wound healing assay.*p < 0.05.

### LOC285194 inhibits invasion and migration by targeting S100A4 expression *in vitro*

To determine the mechanism of action by which LOC285194 influences the migration and invasion of A7r5 cells, S100A4 expression was examined. S100A4 protein expression was blocked in the LOC285194-overexpressing A7r5 cells compared with Lv-NC and negative control transfected A7r5 cells (Figure 3(b)). To confirm that LOC285194-mediated inhibition of migration and invasion is indeed linked to S100A4, the cells were transfected with S100A4 siRNA for 24 h, which significantly inhibit S100A4 expression by western blot assay (Figure 3(c)). Figure 4(d) shows that targeting S100A4 reduced A7r5 cell migration and invasion by wound healing assays. And targeting S100A4 also decreased cell invasion and migration by the Transwell assay (Figure 3(e,f)). We next examined whether LOC285194 affects the expression of MMP-2 and MMP-9,which is associated with cell invasion and migration, only to find that LOC285194 overexpression did not affect MMP-2 and MMP-9 expression in the A7r5 cells (Figure 3(b)).

### LOC285194 targets miR-211 to activates PUMA pathway

**Figure 5.**
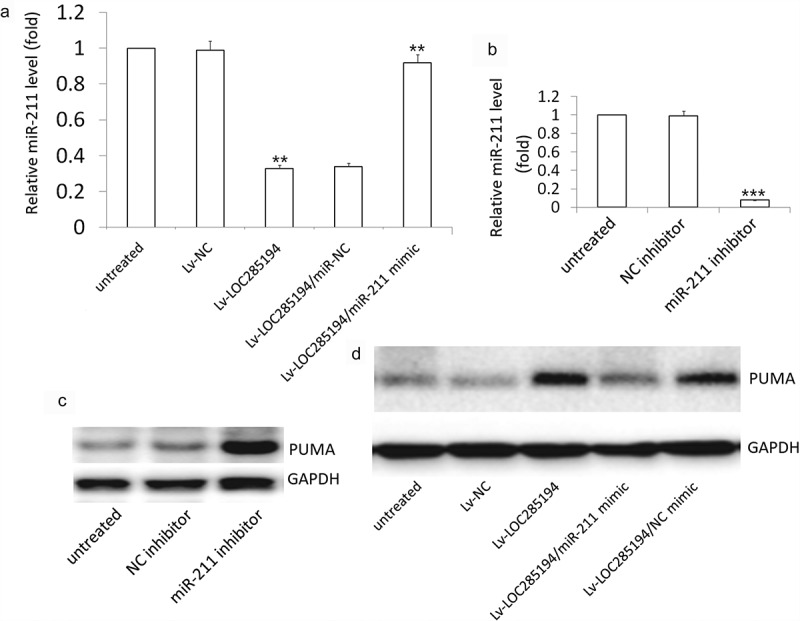
Effect of miR-211 on LOC285194 -induced PUMA expression.

Lv-LOC285194 treatment repressed the expression of miR-211 in the A7r5 cells (Figure 5(a)). The expression of PUMA was increased in the miR-211 inhibitor-treated A7r5 cells compared with the control miR-NC treated A7r5 cells (Figure 5(b,c)). PUMA expression was reversed in LOC285194 transfected A7r5 cells after transfection with miR-211 mimic (Figure 5(d)). These results indicated that LOC285194 upregulated PUMA by blocking miR-211.

(a) The A7r5 cells were transfected with Lv-LOC285194 or co-transfected with miR-211 mimic or its control miR-NC for 72 h. miR-211 expression was detected by qPCR assay; (b) The A7r5 cells were transfected with miR-211 inhibitor or its control NC inhibitor for 72 h. miR-211 expression was detected by qPCR assay; (c) PUMA protein expression was detected by western blot assay. (d) The A7r5 cells were transfected with Lv-LOC285194 or co-transfected with miR-211 mimic or its control miR-NC for 72 h. PUMA protein expression was detected by western blot assay.

### LOC285194 targets TGF-β1 to blocks S100A4 expression

**Figure 6.**
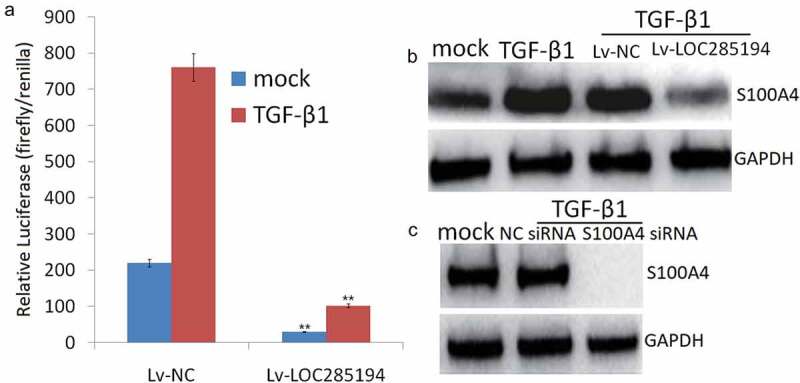
TGF-β1 is required for S100A4 inhibition by LOC285194.

Based on the aforementioned observation that LOC285194 could repress the expression of S100A4, we asked whether the down-regulation of S100A4 by LOC285194 was dependent on the induction of TGF-β inactivity. The luciferase activity was dramatically increased in TGF-β1-treated A7r5 cells, but this effect was reversed in LOC285194-expressing A7r5 cells (Figure 6(a)). The expression of S100A4 was also increased in TGF-β1-treated A7r5 cells but significantly decreased after transfection with LOC285194 (Figure 6(b)). The knockdown of S100A4 also inhibited TGF-β-mediated S100A4 upregulation (Figure 6(c)), suggesting that LOC285194 blocks TGF-β signaling and inhibits S100A4 upregulation.

(a) A7r5 cells were Lv-LOC285194 or Lv-NC (control) and then treated with TGF-β1 (5 ng/ml; 24 h) (+) or mock treated (-), Luciferase is expressed as a ratio of firefly/renilla. (b) S100A4 was assessed by Western blot. (c) A7r5 cells were transfected with S100A4 siRNA or control siRNA for 48 h, then treated with TGF-β1 (5 ng/ml; 24 h), S100A4 was assessed by Western blot.**p < 0.01.

## Discussion

Vascular smooth muscle cells (VSMC) are fully differentiated cells, exhibiting a very low rate of proliferation in healthy mature blood vessels. Smooth muscle α‐actin (α-SM) is richly expressed in the VSMC cells to maintain vessel tone, blood pressure and blood flow [22]. Following vascular injury or in association with a variety of diseases, VSMC exhibit a decrease in expression of differentiation markers and acquire a proliferative phenotype characterized by enhanced cell proliferation and migration [23]. The abnormal proliferation and migration of vascular smooth muscle cell (VSMC) are key components of various vascular diseases, including hypertension, atherosclerosis and vascular stenosis after vessel transplant [24,25]. In addition, VSMC apoptosis is one of the dominant factors that induces plaque vulnerability in atherosclerosis [26]. Therefore, understanding the mechanisms that control normal SMC phenotypic switch is likely to provide key insights toward knowing the biology of atherosclerosis and the development of new therapeutic targets. Our current study is focused on the roles of LOC285194 overexpression in suppressing VSMC proliferation, migration and induced apoptosis in a rat aortic VSMC A7r5 cells.

VSMC apoptosis may be a central event in plaque rupture and its subsequent sequelae [27]. VSMC apoptosis is one of the dominant factors that induces plaque vulnerability in atherosclerosis. Indeed, symptomatic plaques exhibit increased levels of VSMC apoptosis [27] compared with stable lesions. Identifying the mechanisms that regulate VSMC apoptosis potentially may lead to novel therapeutic approaches that are more efficacious for these patients. Previous works showed that LOC285194 overexpression induced cell apoptosis in gastric carcinoma [28], pancreatic cancer [29] and colon cancer [10]. In the present study, we demonstrated that LOC285194 overexpression significantly induced A7r5 cell apoptosis. The abnormal proliferation of vascular smooth muscle cell (VSMC) is the key component of various vascular diseases, including hypertension, atherosclerosis and vascular stenosis after vessel transplant. In this study, LOC285194 overexpression significantly decreased cell proliferation in A7r5 cells *in vitro*, which was agreed with the previous report in osteosarcoma [30] and colon cancer [10].

PUMA (p53 upregulated modulator of apoptosis) is a pro-apoptotic gene, which inhibits all the five anti-apoptotic proteins (Mcl-1, Bcl-2, Bcl-XL, Bcl-W and A1) and directly triggers apoptosis mediated by pro-apoptotic proteins Bax/Bak [31]. PUMA is induced by a wide variety of stimuli, including genotoxic stress, deregulated oncogene expression, toxins, altered redox status, growth factor/cytokine withdrawal and infection [32]. In this study, we firstly demonstrated that LOC285194 overexpression upregulated PUMA expression in A7r5 cells *in vitro*. LOC285194 overexpression-mediated anti-proliferation and pro-apoptosis effects were significantly blocked with PUMA downregulation in A7r5 cells.

Emerging evidence shows that miRNAs exert functions by regulating translation or stability of target mRNAs. In this study, LOC285194 overexpression downregulated miR-211 expression in the A7r5 cells. miR-211 overexpression by miR-211 mimic transfection also resulted in a dramatic suppression of apoptosis and promotion of proliferation in LOC285194-overexpressing A7r5 cells, followed by decreased PUMA expression. Similarly, miR-211 downregulation by miR-211 inhibitor transfection facilitated apoptosis and lowered proliferation of VSMCs followed by increased PUMA expression. We, therefore, concluded that loc285194 induces VSMC cell apoptosis and lower cell proliferation by miR-211-PUMA signal pathway.

The presence of a large number of intimal VSMCs, for example, forming a fibrous cap, has been taken as evidence that VSMC migration from the media plays an important role in atherogenesis. Inhibiting both the migration of VSMCs and the thickening of the intima from the molecular level may be beneficial to prevent arteriosclerosis [33]. Previous studies have demonstrated that S100A4 stimulated migration in human pulmonary artery smooth muscle cell [34] and vascular smooth muscle cell [35]. Furthermore, S100A4 expression was closely correlated with increased migration ability and EMT process induced by TGF-β stimulation [36]. In this study, LOC285194 overexpression inhibits invasion and migration in A7r5 cells *in vitro* via inhibiting TGF-β activity and S100A4 expression. Activation of TGF-β restored LOC285194 overexpression-induced migration inhibition and S100A4 expression. We further validated by luciferase assay that TGF-β1 was regulated by LOC285194. The pre-experimental study found that the treatment of A7r5 cells with miR-211 mimic or inhibitor did not affect S100A4 expression or LOC285194-induced S100A4 expression, suggesting that miR-211 did not relate with LOC285194-induced S100A4 expression. We, therefore, concluded that LOC285194 inhibits invasion and migration in A7r5 cells by TGF-β1-S100A4 signal pathway.

MMPs are important regulators of the vascular ECM and other signaling pathways in the vasculature. Activities of the matrix metalloproteinases (MMPs) MMP-2 and MMP-9 involve the proliferation and migration of vascular smooth muscle cells (VSMCs) [37]. However, in this study, LOC285194 overexpression did not affect MMP-2 and MMP-9 expression. Whether LOC285194 is involved in other MMP regulation need further investigation.

## Conclusions

Collectively, this study shows that LOC285194 inhibits migration and proliferation of VSMCs, and facilitates cell apoptosis. LOC285194 suppressed miR-211 and induced PUMA expression. Targeting PUMA prevents the damaging effects of LOC285194 on A7r5 cells. LOC285194 inhibits invasion and migration of VSMCs via targeting TGF-β1/S100A4 signal. These effects demonstrating for the first time that LOC285194 overexpression may become a promising treatment option in the prevention of atherosclerosis.

## Research highlights

1. LOC285194 inhibited cell proliferation, invasion and migration, and promoted cell apoptosis.
2. LOC285194 inhibits proliferation and promoted apoptosis via targeting miR-211/PUMA signal.
3. LOC285194 decreased cell invasion and migration by targeting TGF-β1/S100A4 signal

## References

[1] Jensen RV, Hjortbak MV, Bøtker HE. Ischemic heart disease: an update. Semin Nucl Med. 2020;50(3):195–207. 32284106 10.1053/j.semnuclmed.2020.02.007

[2] Robinson JG, Huijgen R, Ray K, et al. Determining when to add nonstatin therapy: a quantitative approach. J Am Coll Cardiol. 2016;68(22):2412–2421. 27908345 10.1016/j.jacc.2016.09.928

[3] Hassanin AM, Abdel-Hamid AZ. Cavernous smooth muscles: innovative potential therapies are promising for an unrevealed clinical diagnosis. Int Urol Nephrol. 2020;52(2):205–217. 31617065 10.1007/s11255-019-02309-9

[4] Maranhão RC, Leite AC Jr. Development of anti-atherosclerosis therapy based on the inflammatory and proliferative aspects of the disease. Curr Pharm Des. 2015;21(9):1196–1204. 25312729 10.2174/1381612820666141013150714

[5] Evan G, Littlewood T. A matter of life and cell death. Science. 1998;281(5381):1317–1322. 9721090 10.1126/science.281.5381.1317

[6] Andrés V, Pello OM, Silvestre-Roig C. Macrophage proliferation and apoptosis in atherosclerosis. Curr Opin Lipidol. 2012;23(5):429–438. 22964992 10.1097/MOL.0b013e328357a379

[7] Chong H, Wei Z, Namuhan SG, et al. The PGC-1α/NRF1/miR-378a axis protects vascular smooth muscle cells from FFA-induced proliferation, migration and inflammation in atherosclerosis. Atherosclerosis. 2020;297:136–145. 32120345 10.1016/j.atherosclerosis.2020.02.001

[8] Chen LD, Zhu WT, Cheng YY, et al. T-cell death-associated gene 8 accelerates atherosclerosis by promoting vascular smooth muscle cell proliferation and migration. Atherosclerosis. 2020;297:64–73. 32078831 10.1016/j.atherosclerosis.2020.01.017

[9] Cheng Q, Zhang M, Zhang M, et al. Long non-coding RNA LOC285194 regulates vascular smooth muscle cell apoptosis in atherosclerosis. Bioengineered. 2020;11(1):53–60. 31884873 10.1080/21655979.2019.1705054 PMC6961585

[10] Liu Q, Huang J, Zhou N, et al. LncRNA loc285194 is a p53-regulated tumor suppressor. Nucleic Acids Res. 2013;41(9):4976–4987. 23558749 10.1093/nar/gkt182 PMC3643595

[11] Liu W, Miao Y, Zhang L, et al. MiR-211 protects cerebral ischemia/reperfusion injury by inhibiting cell apoptosis. Bioengineered. 2020;11(1):189–200. 32050841 10.1080/21655979.2020.1729322 PMC7039642

[12] Sun Y, Ye P, Wu J, et al. Inhibition of intimal hyperplasia in murine aortic allografts by the oral administration of the transforming growth factor-beta receptor I kinase inhibitor SD-208. J Heart Lung Transplant. 2014;33(6):654–661. 24685405 10.1016/j.healun.2014.02.020

[13] Nuessle JM, Giehl K, Herzog R, et al. TGFβ1 suppresses vascular smooth muscle cell motility by expression of N-cadherin. Biol Chem. 2011;392(5):461–474. 21375457 10.1515/BC.2011.053

[14] Guo J, Chen H, Ho J, et al. TGF-beta-induced GRK2 expression attenuates AngII-regulated vascular smooth muscle cell proliferation and migration. Cell Signal. 2009;21(6):899–905. 19385060 10.1016/j.cellsig.2009.01.037

[15] Ambartsumian N, Klingelhöfer J, Grigorian M. The Multifaceted S100A4 Protein in Cancer and Inflammation. Methods Mol Biol. 2019;1929:339–365. 30710284 10.1007/978-1-4939-9030-6_22

[16] Fei F, Qu J, Li C, et al. Role of metastasis-induced protein S100A4 in human non-tumor pathophysiologies. Cell Biosci. 2017;7(1):64. 29204268 10.1186/s13578-017-0191-1 PMC5702147

[17] Xie R, Schlumbrecht MP, Shipley GL, et al. S100A4 mediates endometrial cancer invasion and is a target of TGF-β1 signaling. Lab Invest. 2009;89(8):937–947. 19506550 10.1038/labinvest.2009.52 PMC2718065

[18] Matsuura I, Lai CY, Chiang KN. Functional interaction between Smad3 and S100A4 for TGF-beta-mediated cancer cell invasiveness. Biochem J. 2010;426(3):327–335. 20070253 10.1042/BJ20090990

[19] Kitao A, Sato Y, Sawada-Kitamura S, et al. Endothelial to mesenchymal transition via transforming growth factor-beta1/Smad activation is associated with portal venous stenosis in idiopathic portal hypertension. Am J Pathol. 2009;175(2):616–626. 19608867 10.2353/ajpath.2009.081061 PMC2716961

[20] Cunningham MF, Docherty NG, Burke JP, et al. S100A4 expression is increased in stricture fibroblasts from patients with fibrostenosing Crohn’s disease and promotes intestinal fibroblast migration. Am J Physiol Gastrointest Liver Physiol. 2010;299(2):G457–466. 20489045 10.1152/ajpgi.00351.2009

[21] Wang H, Shi J, Luo Y, et al. Long noncoding RNA loc285194 expression in human papillomavirus-positive and -negative cervical squamous cell carcinoma, C33A, and SiHa cells and transforming growth factor-β1. Clin Cancer Res. 2014;20(22):5835–5847. 31774069 10.12659/MSM.917763 PMC6898980

[22] Qi Y, Dai F, Gu J, et al. Biomarkers in VSMC phenotypic modulation and vascular remodeling. Die Pharmazie. 2019;74(12):711–714. 31907108 10.1691/ph.2019.9743

[23] Afra S, Matin MM. Potential of mesenchymal stem cells for bioengineered blood vessels in comparison with other eligible cell sources. Cell Tissue Res. 2020;380(1):1–13. 31897835 10.1007/s00441-019-03161-0

[24] Wang D, Uhrin P, Mocan A, et al. Vascular smooth muscle cell proliferation as a therapeutic target. Part 1: molecular targets and pathways. Biotechnol Adv. 2018;36(6):1586–1607. 29684502 10.1016/j.biotechadv.2018.04.006

[25] Shi N, Chen SY. Smooth muscle cell differentiation: model systems, regulatory mechanisms, and vascular diseases. J Cell Physiol. 2016;231(4):777–787. 26425843 10.1002/jcp.25208

[26] Grootaert MOJ, Moulis M, Roth L, et al. Vascular smooth muscle cell death, autophagy and senescence in atherosclerosis. Cardiovasc Res. 2018;114(4):622–634. 29360955 10.1093/cvr/cvy007

[27] Bennett MR, Sinha S, Owens GK. Vascular smooth muscle cells in atherosclerosis. Circ Res. 2016;118(4):692–702. 26892967 10.1161/CIRCRESAHA.115.306361 PMC4762053

[28] Zhong B, Wang Q, He J, et al. LncRNA LOC285194 modulates gastric carcinoma progression through activating Wnt/β-catenin signaling pathway. Cancer Med. 2020;9(6):2181–2189. 31991056 10.1002/cam4.2844 PMC7064030

[29] Wang H, Jiao H, Jiang Z, et al. Propofol inhibits migration and induces apoptosis of pancreatic cancer PANC-1 cells through miR-34a-mediated E-cadherin and LOC285194 signals. Bioengineered. 2020;11(1):510–521. 32303144 10.1080/21655979.2020.1754038 PMC7185861

[30] Pasic I, Shlien A, Durbin AD, et al. Recurrent focal copy-number changes and loss of heterozygosity implicate two noncoding RNAs and one tumor suppressor gene at chromosome 3q13.31 in osteosarcoma. Cancer Res. 2010;70(1):160–171. 20048075 10.1158/0008-5472.CAN-09-1902

[31] Yu J, Wang Z, Kinzler KW, et al. PUMA mediates the apoptotic response to p53 in colorectal cancer cells. Proc Natl Acad Sci U S A. 2003;100(4):1931–1936. 12574499 10.1073/pnas.2627984100 PMC149936

[32] Yu J, Zhang L. PUMA, a potent killer with or without p53. Oncogene. 2008;27(Suppl 1):S71–83. 19641508 10.1038/onc.2009.45 PMC2860432

[33] Tinajero MG, Gotlieb AI. Recent developments in vascular adventitial pathobiology: the dynamic adventitia as a complex regulator of vascular disease. Am J Pathol. 2020;190(3):520–534. 31866347 10.1016/j.ajpath.2019.10.021

[34] Spiekerkoetter E, Lawrie A, Merklinger S, et al. Mts1/S100A4 stimulates human pulmonary artery smooth muscle cell migration through multiple signaling pathways. Chest. 2005;128(6):577S. 10.1378/chest.128.6_suppl.577S 16373840

[35] Spiekerkoetter E, Guignabert C, de Jesus Perez V, et al. S100A4 and bone morphogenetic protein-2 codependently induce vascular smooth muscle cell migration via phospho-extracellular signal-regulated kinase and chloride intracellular channel 4. Circ Res. 2009;105(7):639–647. 19713532 10.1161/CIRCRESAHA.109.205120 PMC2818124

[36] Li F, Shi J, Xu Z, et al. S100A4-MYH9 axis promote migration and invasion of gastric cancer cells by inducing TGF-β-mediated epithelial-mesenchymal transition. J Cancer. 2018;9(21):3839–3849. 30410586 10.7150/jca.25469 PMC6218764

[37] Yu MH, Lin MC, Huang CN, et al. Acarbose inhibits the proliferation and migration of **vascular smooth muscle cells** via targeting Ras signaling. Vascul Pharmacol. 2018;103-105:8–15. 29432898 10.1016/j.vph.2018.02.001

